# Rectosigmoidal manifestations of venous malformations: MR imaging findings and interdisciplinary therapeutic modalities

**DOI:** 10.1038/s41598-019-56217-0

**Published:** 2019-12-27

**Authors:** Richard Brill, Eva Brill, Wibke Uller, Veronika Teusch, Hubert Gufler, Simone Hammer, Claudia Fellner, Katja Evert, Constantin Goldann, Maximilian Helm, Rosendahl Jonas, Walter A. Wohlgemuth

**Affiliations:** 1 Universitätsklinikum Halle (Saale), Martin-Luther-Universität Halle-Wittenberg, Department of Radiology and Policlinic of Radiology, Ernst-Grube-Straße 40, D-06120 Halle (Saale), Germany; 2 Familienpraxis im Hof, Department of General Medicine, Innere Neumarkter Str. 2a, D- 84453 Mühldorf, Germany; 3 Universitätsklinikum Regensburg, Universität Regensburg, Department of Radiology, Franz-Josef-Strauß-Allee 11, D-93053 Regensburg, Germany; 4 Universitätsklinikum Regensburg, Universität Regensburg, Department of Pathology, Franz-Josef-Strauß-Allee 11, D-93053 Regensburg, Germany; 5 Universitätsklinikum Halle (Saale), Martin-Luther-Universität Halle-Wittenberg, Department of Gastroenterology, Ernst-Grube-Straße 40., D-06120 Halle (Saale), Germany

**Keywords:** Lower gastrointestinal bleeding, Outcomes research

## Abstract

The aim of this study was to identify the frequency of rectosigmoidal involvement in patients with venous malformations (VM) of the lower extremities and to demonstrate multidisciplinary therapeutic options. The medical records and magnetic resonance images (MRI) of patients with VM of the lower extremities, over a six-year period, were reviewed retrospectively in order to determine the occurrence of rectosigmoidal involvement. Vascular interventions, surgical treatments, percutaneous and hybrid (endoscopy-guided angiography) sclerotherapy and procedural complications (according to Clavien-Dindo classification) were also noted. Of the 378 patients with vascular malformation of the lower limbs, 19 patients (5%) had documented venous rectosigmoidal malformation. All of these 19 patients reported episodes of rectal bleeding, while seven patients (36.8%) also had anemia. All patients underwent endoscopy. By endoscopy, seven patients (36.8%) showed discreet changes, and 12 patients (63.2%) showed pronounced signs of submucosal VM with active (47.3%) or previous (15.7%) bleeding. Treatment was performed in all patients with pronounced findings. Six patients underwent endoscopy-guided hybrid sclerotherapy, one patient underwent endoscopic tissue removal, one patient received percutaneous sclerotherapy and one patient received a combination of transvenous embolization and hybrid sclerotherapy. Three patients required open surgery. No complications occurred after conservative treatments; however, one complication was reported after open surgery. None of the treated patients reported further bleeding and anemia at the end of the follow-up period. In this cohort, rectosigmoidal VM occurred in 5% of patients presenting with a high incidence of rectal bleeding. Percutaneous or endoscopy-guided hybrid sclerotherapy appeared to be a safe and effective alternative to surgery.

## Introduction

Anorectal bleeding is the most common indication for gastrointestinal endoscopy; however, the underlying pathologies are variable^1^. Although gastrointestinal tumors are frequent, rare pathologies like congenital vascular anomalies must be kept in mind. Vascular anomalies are subclassified into tumors and malformations according to ISSVA classification^2^. Tumors arise out of endothelial cell proliferation whereas VMs are mesenchymal and angiogenic disorders of the vessel wall and occur during embryogenesis^3^. Although tumors can diminish with lifetime, VMs grow consecutively^4^. Unfortunately, the ISSVA classification is often not used, leading to confusing nomenclature^5^. Literature about hemangiomas is more common, as this type of tumor not only has a higher incidence, but is also used as a diagnosis in a broader and often more imprecise manner. In the current study, VM was strictly defined by the ISSVA classification.

In addition to venous origin, malformations can also appear as capillary, lymphatic, arterio-venous and combined malformations; however, VMs are the most common type of malformation comprising two-thirds of all congenital vascular malformations^6^. In regard to flow characteristics, VMs are part of the subgroup of slow-flow malformations, that also consists of lymphatic and capillary malformations, and their combinations. Arterio-venous malformations are high-flow malformations. These flow characteristics are important as they predefine the treatment options^7^.

40% of VMs are located in the head and neck region, 40% in the extremities and 20% involve the trunk^8^. VMs of the gastrointestinal tract are rare and can affect all parts of the intestinal digestive system, but are often located in the rectosigmoidal area^9^. Although bleeding can be occult or visible^9^, these lesions often have a pathognomonic appearance as bluish vascular convolutes, but can be difficult to detect and require a multidisciplinary approach for treatment^5,10^. Endoscopy is used to show the intramucosal and intraluminal part of the lesion and the origin of the hemorrhage. MRI has shown high sensitivity and specificity for illustrating the extension and depth of involvement of the gastrointestinal wall^11^, as well as the surrounding tissue.

The primary purpose of this study was to determine the frequency of rectosigmoidal involvement in patients with VMs of the lower limbs using MRI. The secondary purpose was to correlate the MRI findings with endoscopy and with clinically relevant findings such as bleeding and anemia. The tertiary purpose was to discuss different options for endoscopic and interventional radiological therapies in a structured interdisciplinary environment.

## Material and Methods

### Study population

All MR images of patients with Vascular Malformations of the lower extremities at the University of Regensburg, Germany from 2011 to 2017 were evaluated in order to detect accompanying occurrences of rectosigmoidal manifestations of VMs. This study refers to VMs only as defined by the International Society for Study of Vascular Anomalies (ISSVA) classification^2^. Institutional review board (University of Regensburg) approval was obtained prior to data collection and all methods were performed in accordance with relevant guidelines and regulations (ethics vote Number 18-886-104). Participation in this study was voluntary and informed consent was obtained from all participants. For patients under the age of 18 years, a parent or legal guardian gave informed consent. Episodes of bleeding and anemia (hemoglobin of less than 13 g/dl in men, 12 g/dl in women) were recorded for all patients with rectosigmoidal involvement via patient documentation and anamnesis. Exclusion criteria comprised malformations associated with other anomalies. Endoscopic examination, as well as endoscopic treatment and surgical treatment data were recorded for all patients suffering from rectosigmoidal venous malformation manifestations.

### Acquisition of MRI and definition of colorectal involvement

A 3.0 Tesla scanner (Skyra, Siemens Healthcare, Erlangen, Germany) was used for bilateral large-field-of-view (FoV) MRI of the pelvis and legs. Depending on the location of the VM, imaging from the pelvis to the feet was performed with array coils. Different sequences were used to delineate VM and surrounding tissues. A standardized MRI protocol was used and contained following sequences: Axial T2-weighted turbo-spin echo (8 mm), short tau inversion recovery (STIR) for axial (8 mm) and coronal (6 mm), T1-weighted turbo-spin echo coronal (6 mm) before and T1 weighted high-resolution 3D gradient echo with spectral fat saturation (volumetric interpolated breath hold examination, VIBE) (0,8 mm) after injection of contrast medium, and time-resolved MR- angiography with interleaved stochastic trajectories (TWIST) with high temporal resolution. The field of view included the complete pelvis from sigmoid to anus. Criteria for a VM involving the rectosigmoidal region were hyperintense masses in T2 sequences around or in the gastrointestinal wall, wall-thickening, and increased signal intensity. Following injection of Gadolinium contrast medium, VMs show enhancement on T1 weighted sequences.

### Multidisciplinary treatments and follow-up

**Figure 1:**
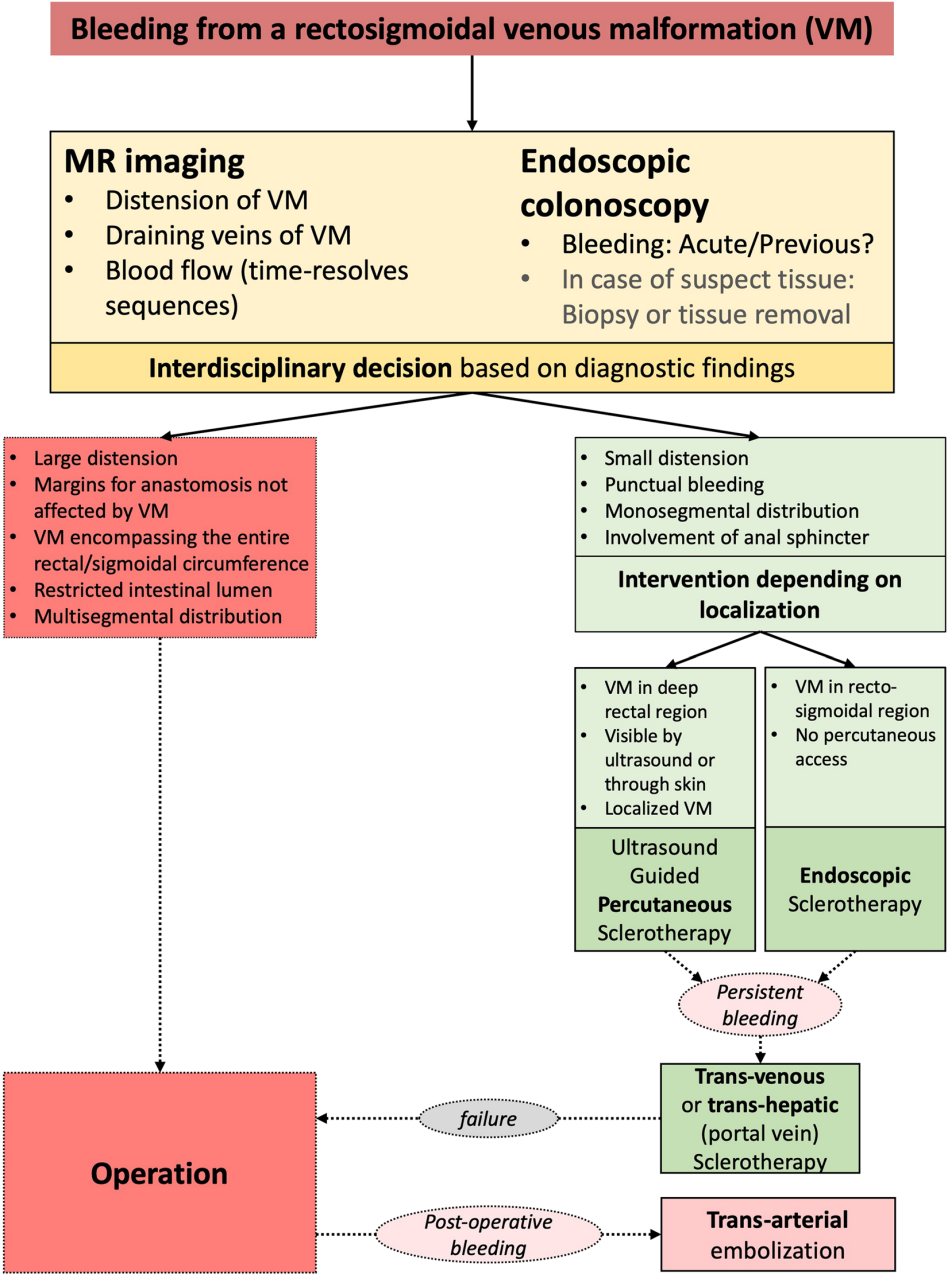
Therapeutic algorithm for the treatment of bleeding from a VM.

There are several treatment modalities used for rectosigmoidal VMs. These treatments are often aimed at reducing or removing the rectosigmoidal malformation, as well as ameliorating or stopping symptoms, such as bleeding and anemia. The patients used in this study received angiographic transvenous embolization with coils and plugs, percutaneous and endoscopy-guided hybrid sclerotherapy with Polidocanol (3% Aethoxysklerol®, Kreussler & Co. GmbH, Wiesbaden, Germany, 1:4 prepared with the Easy Foam Kit®, Kreussler & Co. GmbH, Wiesbaden, Germany) or colorectal surgery. The specific treatment used was determined by evaluation of morphological characteristics and extension of the VM. Indications for different therapy modalities to control bleeding are described in Fig. 1.

The postoperative/postinterventional complications were analyzed according to the Clavien-Dindo Classification^12^.

To evaluate the benefits of the therapeutic intervention, patients were followed up via outpatient consultation. There was a total of three consultations: two in the first twelve months and then a third two years after therapy. Episodes of macroscopic bleeding were specifically investigated. Hemoglobin was checked at the time of MRI examination, endoscopy and during follow-up.

## Results

378 patients suffering from a vascular malformation of the lower extremity as diagnosed at the Interdisciplinary Tertiary Care Vascular Anomaly Center were included with available pelvic MR imaging. After analyzing the MRIs of all 378 patients, 19 patients showed rectosigmoidal VM (5.0%). 10 patients were female, 9 were male. These patients’ mean age was 20 years (min. 4, max. 55; standard variance 14). 31% were children under 18 years (min. 4, max. 10, mean age 6 years).

**Figure 2:**
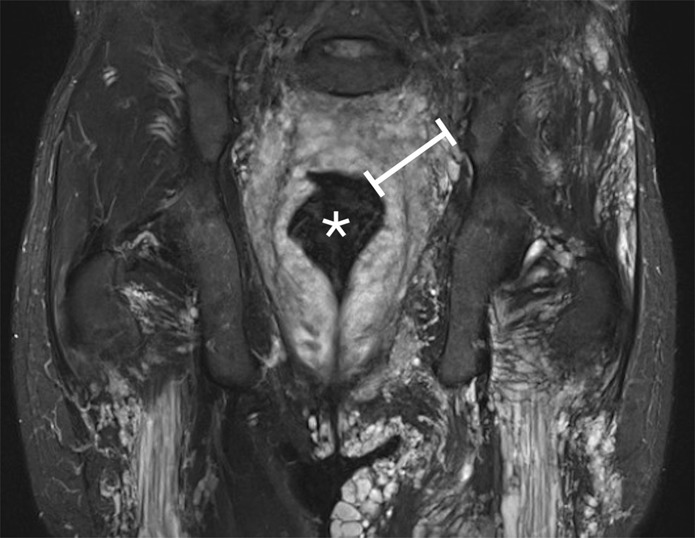
MRI of a 35-year old patient with expanded VM, both rectosigmoidal and in lower limbs. (*) Rectosigmoidal lumen; (I) rectosigmoidal intramural VMs associated with massively dilated rectosigmoidal wall.

**Figure 3:**
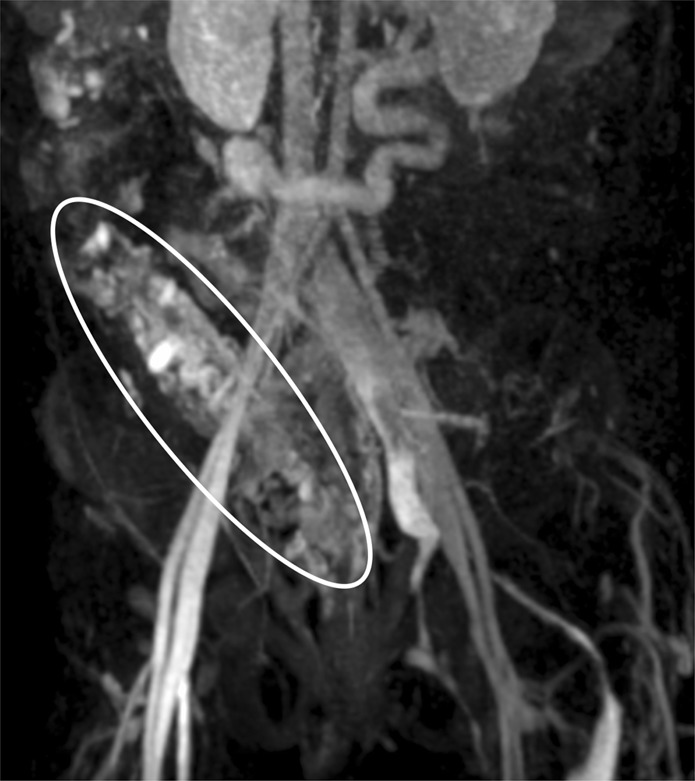
TWIST MRI of a 10-year old girl with expanded sigmoidal VM. Circle: Massively dilated vessels with venous flow characteristics.

The localization, affected side, associated anomalies and the extension of the rectosigmoidal involvement, as well as the endoscopic reports are summarized in Table 1. Figures 2 and 3 show MR images of a 35-year old and 10-year old patient, respectively. In a detailed documented anamnesis, all patients confirmed frequent and extensive episodes of rectal macroscopic visible bleeding. All 19 patients underwent coloscopy. No patient showed invasion of the anal sphincter and the maximal extension was up to the *linea dentata*. Seven patients showed discreet changes compared to normal coloscopic findings, while 12 patients showed pronounced signs of VM with active (9) or previous (3) bleeding. Treatment was performed in all 12 of these patients.

**Table 1:** Patient information.

patient	localization/quadrant of VM	Syndrome	side (b = bilateral r = right l = left)	MRI	Endoscopy report
1	lower	yes	b	VM Rectum	pronounced signs (filling the whole rectum, lumen restricting, active bleeding)
2	lower	no	l	VM Rectum	pronounced signs (punctuated increased vascular network in the anal canal with bleeding)
3	lower	no	l	VM Sigma, Rectum	pronounced signs (extensive, spot-like bleeding, elongated)
4	upper + lower	yes	b	VM Sigma, Rectum	discreet signs (increased vascular network)
5	lower	no	l	VM Sigma, Rectum	discreet signs (increased vascular network, elongated)
6	upper + lower	yes	b	VM Sigma, Rectum	pronounced signs (elongated, punctuated bleeding)
7	upper + lower	yes	b	VM Sigma, Rectum	pronounced signs (extensive, elongated, whole circumference, lumen restricting, multiple spots of previous bleeding)
8	upper + lower	yes	b	VM Sigma, Rectum	pronounced signs (active bleeding, segmental)
9	lower	yes	l	VM Rectum	discreet signs (increased vascular network, segmental)
10	lower	yes	b	VM Sigma	pronounced signs (active bleeding, segmental, lumen restricting)
11	lower	yes	b	VM Sigma, Rectum	pronounced signs (active bleeding over a long distance)
12	lower	no	r	VM Rectum	discreet signs (increased vascular network, segmental)
13	lower	no	b	VM Sigma, Rectum	pronounced signs (active punctual bleeding, increased vascular network over a long distance)
14	upper + lower	no	b	VM Rectum	pronounced signs (punctuated bleeding, malignancy suspected tissue)
15	lower	no	l	VM Rectum	discreet signs (increased vascular network, segmental)
16	upper + lower	no	b	VM Rectum	pronounced signs (signs of previous bleeding, spot-like)
17	lower	yes	b	VM Rectum	discreet signs (increased vascular network, segmental)
18	lower	no	l	VM Rectum	pronounced signs (signs of previous bleeding, spot-like)
19	upper + lower	yes	b	VM Rectum	discreet signs (increased vascular network, segmental)

Blood testing at the time of endoscopy revealed anemia in 58.3% of the patients. 12 patients (63.1%) with VM of the rectosigmoid underwent one or several interventional treatments of their lesion. Indications for treatment were determined by signs of active or previous bleeding via coloscopy (Tables 1, 2).

**Table 2:** Treatment.

Pat.	Indication	Treatment	Procedure	Material	Re-Intervention Complications
1	Bleeding, anemia	Operation	Recto sigmoidal resection with protective ileostomy		Angiographic embolization (branch of A. iliaca int.) because of postoperative bleeding (Clavien-Dindo IIIb)
2	Bleeding	Sclerotherapy	Percutaneous	2 ml Polidocanol	No
3	Bleeding, anemia	Sclerotherapy Angiographic embolization	endoscopic transvenous	2 ml Polidocanol 5 detachable interlock coils, 1 Amplatzer vascular plug	Endoscopic, 4 ml Polidocanol
6	Bleeding	Sclerotherapy	endoscopic	3 ml Polidocanol	No
7	Bleeding, anemia	Operation	deep anterior rectal resection, hemorrhoidal-like resection		No
8	Bleeding, anemia	Sclerotherapy	endoscopic	2 ml Polidocanol	No
10	Bleeding	Sclerotherapy	endoscopic	4 ml Polidocanol	No
11	Bleeding, anemia	Operation	deep anterior rectal resection		No
13	Bleeding	Sclerotherapy	endoscopic	3 ml Polidocanol	No
14	Bleeding, anemia, histologic examination of malignancy suspected tissue	Tissue removal	endoscopic	loop	No
16	Bleeding, anemia	Sclerotherapy	endoscopic	2 ml Polidocanol	No
18	Bleeding	Sclerotherapy	endoscopic	3 ml Polidocanol	No

**Figure 4:**
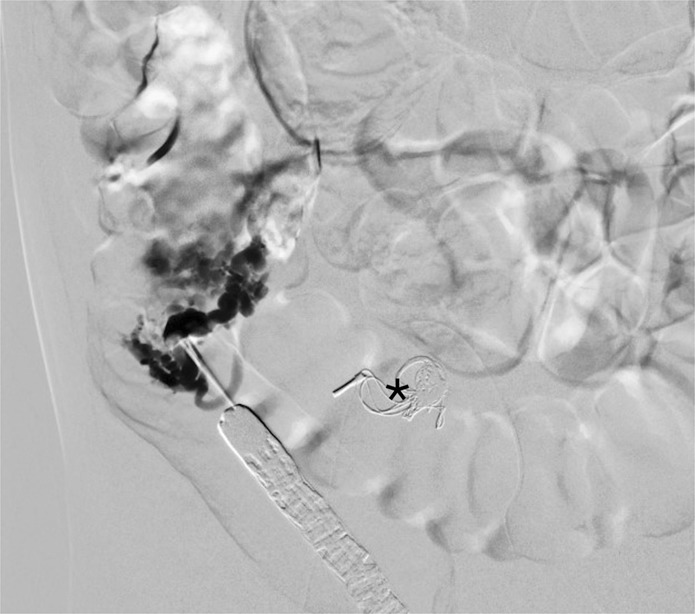
Angiography of a 10-year old girl with VM: Endoscopy-guided puncture of an intramural sigmoidal VM and venous angiography during intervention; (*) coils (transvenous angiographic embolization).

**Figure 5:**
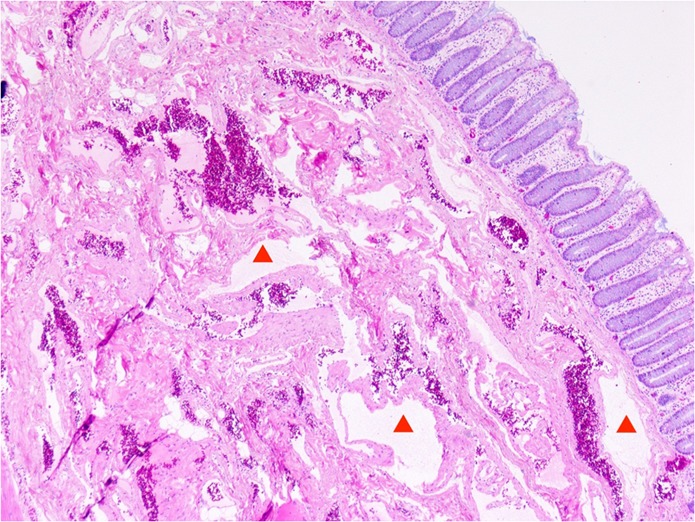
Histologic picture of a 35-year old patient with large VM: HE staining shows dilatated blood vessels (marked with a triangle) with thin walls in the submucosa (original magnification 40×).

A single intervention of sclerotherapy was performed for seven patients. Six of these treatments used endoscopy-guided hybrid sclerotherapy, while the seventh received percutaneous direct-puncture sclerotherapy. In one patient, hybrid sclerotherapy was combined with transvenous angiographic embolization (Fig. 4). One loop-assisted endoscopic tissue removal similar to polypectomy was performed to acquire tissue for histology (Fig. 5) of an unclear polypoid tissue that was bleeding (histology confirmed a VM). Procedures and used materials are described in Table 2.

**Figure 6:**
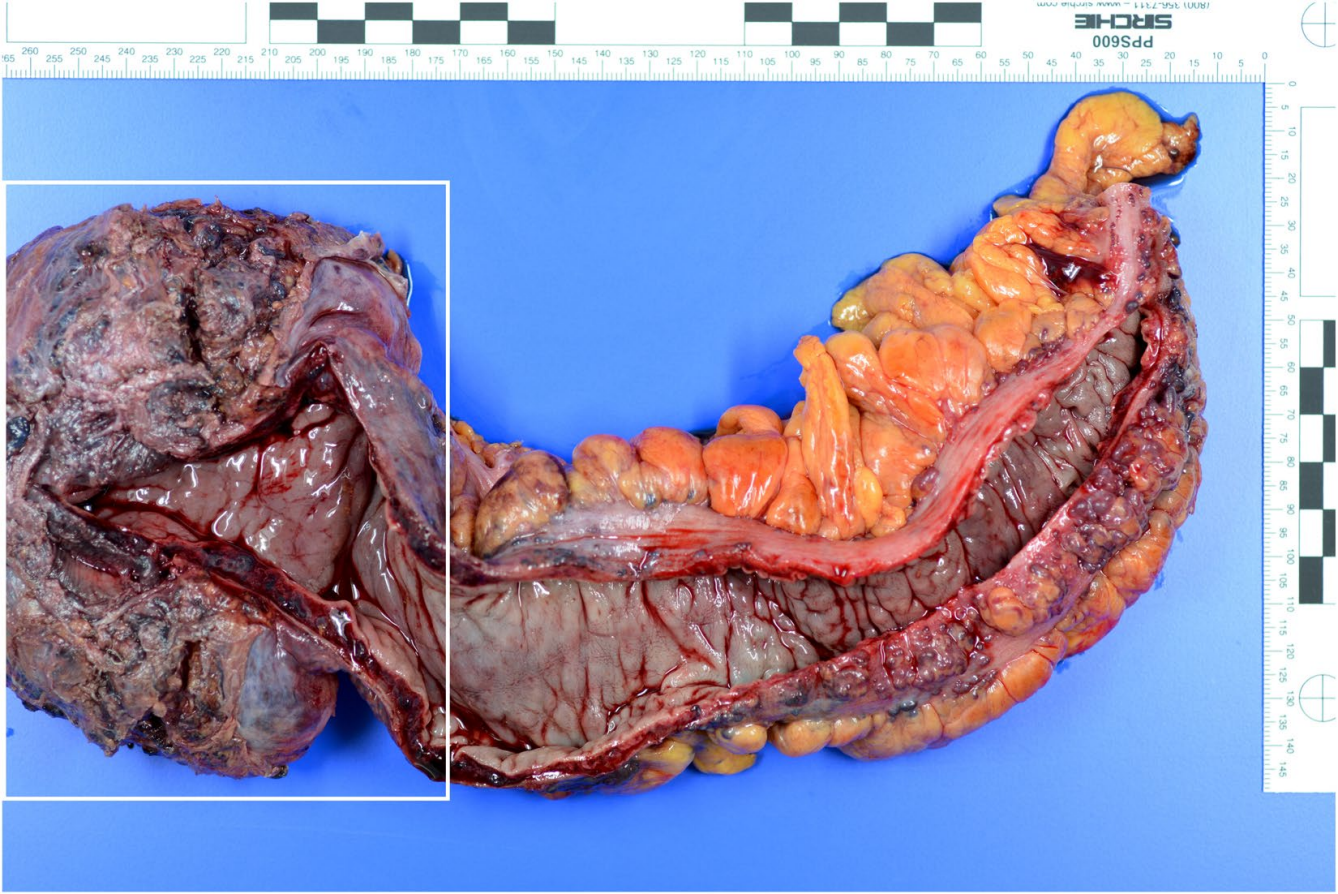
Macroscopic picture of a 35-year old patient with large VM (white rectangle): Gross view of the surgical specimen of a rectum and sigmoid colon with prominent blood vessels, particularly in the surrounding soft tissue.

**Figure 7:**
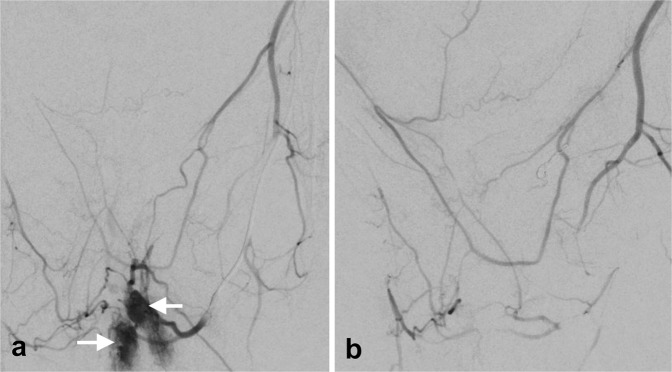
(**a**) Angiography of a 35-year old patient with VM and postoperative bleeding (arrows) out of the inferior rectal artery. (**b**) No bleeding after embolization.

Surgery was necessary for three patients. Among these three patients, two received a deep anterior resection of the rectum with protective ileostomy. In the third patient, the sigmoid and proximal rectum was resected (Fig. 6), which included placement of a protective ileostomy. One of the patients had a history of transrectal hemorrhoidal-like repair due to rectal bleeding. The patient with rectosigmoidal resection needed angiographic embolization because of postoperative bleeding (*A. rectalis inferior*) (Fig. 7a,b) This was the only complication reported (Clavien-Dindo IIIb).

Of the 12 treated patients, only one patient needed reintervention 13 months after initial therapy due to a recurrent episode of bleeding. This patient was treated again with endoscopy-guided hybrid sclerotherapy using 4 ml of Polidocanol and was asymptomatic at the end of the review period. No complications were reported after sclerotherapy. Thus, bleeding episodes and anemia disappeared in all patients by the end of follow-up period (2 years).

## Discussion

This is the first study to report the MRI findings of rectosigmoidal involvement in patients with VM of the lower limbs, as well as the outcomes of conservative and surgical treatments. The incidence of involvement of the lower GI tract in our patients was 5%. We found that approximately 50% of the lesions are located in the rectum only and approximately 50% expanded continuously to the sigmoid, whereas the anal sphincter was spared. MRI was found to be the best imaging modality to detect extent and location of the malformation into the surrounding soft tissues^11^. All 19 patients had a history of bleeding; 12 of them had active or previous bleeding signs (diagnosed by endoscopy) and received treatment (7 sclerotherapy, 1 hybrid intervention, 1 loop-assisted sclerotherapy, 3 surgery). Therefore, a therapeutic algorithm was developed (Fig. 1).

Previous literature has shown that up to 83% of gastrointestinal vascular malformations are known to cause lower gastrointestinal bleeding and are located in the rectum and sigmoid^13,14^. As bleeding is the most common symptom of both hemangiomas and VMs, and because there are various therapies available^15^, an exact diagnosis is critical. Imaging, endoscopy, and interventional radiology are becoming increasingly important in the diagnosis and treatment of colorectal vascular malformations, especially given their morbidity and mortality^16^. In 70% of the cases, endoscopy can reveal the correct diagnosis of a *malformation*^14^ and endoscopic options are often applied to control bleeding^17^.

Intestinal mucosal involvement can be diffuse and extended, but can also appear polypoid^18^, as confirmed in the current study. One of our patients was first diagnosed with a polyp of unknown origin, but after polypectomy, a Capillary Venous Malformation with tissue hyperplasia was diagnosed. Therefore, multifocal involvement of vascular malformations must be expected and sometimes only histology can reveal the underlying pathology.

Sclerotherapy is performed frequently and effectively in the treatment of VMs^19^. Endoscopy-guided hybrid sclerotherapy of colonic VMs may be a viable therapeutic option^5^, but research supporting this treatment is rare and until now has only been described in case reports^20,21^. One study^20^ presented two patients who suffered from massive venous malformations. These two patients had an expanded rectosigmoidal VM with a large extension involving the whole circumference with lumen restriction. These patients were treated with 13 to 15 interventions of hybrid sclerotherapy each. The outcomes were poor, with one patient experiencing decreased, but not cessation, bleeding, and the second patient died due to recurrent bleeding. In our opinion, surgery (if margins for resection/sphincter are free of VM) or a combination of endoscopy and transvenous/transhepatic sclerotherapy (if margins are not free) would have been an adequate therapy because of the immense dimensions of these VMs (Table 1).

Endoscopic colorectal sclerotherapy has been reported as an effective treatment for similar vascular problems (e.g. rectal varices)^22,23^, internal hemorrhoids^24^, or angiodysplasia of the colon^25^. Therapeutic endoscopy offers an effective alternative to surgery for *focal* vascular defects of the colon^26^. In contrast to the results of Amati *et al*., who stated that non-operative therapies like sclerotherapy or interventional angiography result in recurrence of malformations^27^, the current study did not show a high recurrence rate (11%) in patients treated during the follow-up 2 year period.

In the literature, case reports or small case series of laparoscopic or open surgical treatment of vascular malformations of the colon have been reported^13,14,28,29^, but a combination of treatment options or comparison to conservative therapies are lacking. Resection can be a valuable option if the lesion can be eliminated completely^5^. Nevertheless, excision of a VM always bears the risk of severe intraoperative bleeding and injury to adjacent structures, especially in the crowded area of a child’s pelvis. Another important aspect related to the decision for or against open surgical repair is the enlargement of the anal canal, especially the involvement of the anal sphincter. Surgery with a stapled anastomosis should only be performed if the sphincter is not affected. Among the patients in this study, none had invasion of the sphincter as revealed by coloscopy and the maximum extension was up to the *linea dentata*. As such, all of the patients who needed surgical repair were treated in a safe and effective manner.

The primary limitation of the study was the small patient cohort. This small sample size was due to the rare nature of the pathology. Therefore, a multicenter study approach may be necessary for future research.

In summary, percutaneous and endoscopy-guided hybrid sclerotherapy is a good option to treat localized bleeding of VM with less restriction of the intestinal lumen (endoscopic passage possible), although a combination with angiographic methods may be necessary depending on the extension of VM to disconnect from mesenteric blood supply and to avoid recurrence. Surgical treatment should be considered when the VM is large, but the margins of scheduled resection are without pathology (Fig. 7b). For all treatments, risks and benefits should be balanced carefully using an interdisciplinary approach.
