# Author Correction: Rectosigmoidal manifestations of venous malformations: MR imaging findings and interdisciplinary therapeutic modalities

**DOI:** 10.1038/s41598-020-59025-z

**Published:** 2020-02-07

**Authors:** Richard Brill, Eva Brill, Wibke Uller, Veronika Teusch, Hubert Gufler, Simone Hammer, Claudia Fellner, Katja Evert, Constantin Goldann, Maximilian Helm, Jonas Rosendahl, Walter A. Wohlgemuth

**Affiliations:** 1 Universitätsklinikum Halle (Saale), Martin-Luther-Universität Halle-Wittenberg, Department of Radiology and Policlinic of Radiology, Ernst-Grube-Straße 40, D-06120 Halle (Saale), Germany; 2 Familienpraxis im Hof, Department of General Medicine, Innere Neumarkter Str. 2a, D-84453 Mühldorf, Germany; 3 Universitätsklinikum Regensburg, Universität Regensburg, Department of Radiology, Franz-Josef-Strauß-Allee 11, D-93053 Regensburg, Germany; 4 Universitätsklinikum Regensburg, Universität Regensburg, Department of Pathology, Franz-Josef-Strauß-Allee 11, D-93053 Regensburg, Germany; 5 Universitätsklinikum Halle (Saale), Martin-Luther-Universität Halle-Wittenberg, Department of Gastroenterology, Ernst-Grube-Straße 40, D-06120 Halle (Saale), Germany

Correction to: *Scientific Reports* 10.1038/s41598-019-56217-0, published online 27 December 2019

The original version of this Article contained a typographical error in the spelling of the author Jonas Rosendahl, which was incorrectly given as Rosendahl Jonas. This has now been corrected in the PDF and HTML versions of the Article.

